# A novel bifunctional acetyl xylan esterase/arabinofuranosidase from *Penicillium chrysogenum* P33 enhances enzymatic hydrolysis of lignocellulose

**DOI:** 10.1186/s12934-017-0777-7

**Published:** 2017-09-26

**Authors:** Yi Yang, Ning Zhu, Jinshui Yang, Yujian Lin, Jiawen Liu, Ruonan Wang, Fengqin Wang, Hongli Yuan

**Affiliations:** 1 State Key Laboratory of Agrobiotechnology, College of Biological Sciences, China Agricultural University, Beijing, China; 2 College of Life Science, Henan Agricultural University, Zhengzhou, China; 3 National Energy R & D Center for Non-food Biomass, China Agricultural University, Beijing, China

**Keywords:** *Penicillium chrysogenum*, Acetyl xylan esterase, α-l-Arabinofuranosidase, Hydrolysis, Synergy

## Abstract

**Background:**

Xylan, the major constituent of hemicellulose, is composed of β-(1,4)-linked xylopyranosyl units that for the backbone, with side chains formed by other chemical moieties such as arabinose, galactose, mannose, ferulic acid and acetyl groups. Acetyl xylan esterases and α-l-arabinofuranosidases are two important accessory enzymes that remove side chain residues from xylan backbones and may act in synergy with other xylanolytic enzymes. Compared with enzymes possessing a single catalytic activity, multifunctional enzymes can achieve lignocellulosic biomass hydrolysis using a less complex mixture of enzymes.

**Results:**

Here, we cloned an acetyl xylan esterase (PcAxe) from *Penicillium chrysogenum* P33 and expressed it in *Pichia pastoris* GS115. The optimal pH and temperature of the recombinant PcAxe (rPcAxe) for 4-nitrophenyl acetate were 7.0 and 40 °C, respectively. rPcAxe is stable across a broad pH range, retaining 100% enzyme activity om pH 6–9 after a 1 h incubation. The enzyme tolerates the presence of a wide range of metal ions. Sequence alignment revealed a GH62 domain exhibiting α-l-arabinofuranosidase activity with pH and temperature optima of pH 7.0 and 50 °C, in addition to the expected esterase domain. rPcAxe displayed significant synergy with a recombinant xylanase, with a degree of synergy of 1.35 for the hydrolysis of delignified corn stover. Release of glucose was increased by 51% from delignified corn stover when 2 mg of a commercial cellulase was replaced by an equivalent amount of rPcAxe, indicating superior hydrolytic efficiency.

**Conclusions:**

The novel bifunctional enzyme PcAxe was identified in *P. chrysogenum* P33. rPcAxe includes a carbohydrate esterase domain and a glycosyl hydrolase family 62 domain. This is the first detailed report on a novel bifunctional enzyme possessing acetyl xylan esterase and α-l-arabinofuranosidase activities. These findings expand our current knowledge of glycoside hydrolases and pave the way for the discovery of similar novel enzymes.

**Electronic supplementary material:**

The online version of this article (doi:10.1186/s12934-017-0777-7) contains supplementary material, which is available to authorized users.

## Background

Xylan, the major constituent of hemicellulose, is composed of β-(1,4)-linked xylopyranosyl units that for the backbone, with side chains formed by other chemical moieties such as arabinose, galactose, mannose, ferulic acid and acetyl groups [1]. Because of the structural diversity and complexity of xylan, its complete hydrolysis requires a combination of main chain degrading enzymes including xylanases and β-xylosidases, and side chain degrading enzymes comprising α-l-arabinofuranosidases, acetyl xylan esterases, feruloyl esterases and α-glucuronidases [2, 3]. Among these accessory enzymes, acetyl xylan esterases catalyze the cleavage of acetyl substituents from acetylated xylan, and α-l-arabinofuranosidases hydrolyze the glycosidic bonds that link α-l-arabinofuranoyl side chains to the backbone. Therefore, acetyl xylan esterases and α-l-arabinofuranosidases are two important accessory enzymes that remove side chain residues from the hemicellulose backbone and function synergistically with other xylanolytic enzymes [4, 5]. These well-characterized degradative enzymes usually only catalyze a certain reaction.

In order to achieve efficient hydrolysis of lignocellulose, a series of enzymes are required to work in combination, which increases the cost of enzymes used in a biotechnological context. Compared with enzymes possessing a single function, multifunctional enzymes can hydrolyze a variety of different substrates simultaneously, which reduces the amount of enzyme types needed for the hydrolysis of lignocellulose, decreases their cost in biotechnological processes, and has additional advantages. In this sense, multifunctional enzymes are more intriguing than traditional enzymes, especially those involved in heteroxylan hydrolysis [6, 7]. β-d-Xylosidase/α-l-arabinofuranosidase [8], xylanase/α-l-arabinofuranosidase [5, 6] and xylanase/acetyl xylan esterase [9–12] bifunctional systems have been reported. Although the bifunctional α-l-arabinofuranosidase/acetyl xylan esterase was reported in *Arthrobacter* sp. MTCC5214 and *Lactobacillus* sp. based on the elution profile and zymogram analysis [13], a bifunctional enzyme possessing acetyl xylan esterase and α-l-arabinofuranosidase activities has not been studied in detail, and would be potentially useful for hydrolyzing lignocellulose.

*Aspergillus* and *Trichoderma* are two fungal genera that have been widely studied due to their cellulase and hemicellulase activities [14–16]. By contrast, research on *P. chrysogenum* is lacking. The cellulase, xylanase and mannanase activities of *P. chrysogenum* have received some attention [17, 18], but there has been no report of an acetyl xylan esterase from this fungus. We previously isolated the *P. chrysogenum* P33 strain, which is able to degrade lignocellulosic biomass efficiently, and secrete abundant acetyl xylan esterases (unpublished data). In the present study, a bifunctional enzyme from P33 displaying both acetyl xylan esterase and α-l-arabinofuranosidase activities was cloned and expressed in *Pichia pastoris* GS115, and its enzymatic characteristics studied for the first time. The bifunctional enzyme exhibited significant synergy with xylanase, and promoted the hydrolysis of cellulose.

## Methods

### Strains, culture conditions and plasmids

The *P. chrysogenum* P33 (CGMCC 3.15539) used in this study has been maintained in our laboratory since its discovery. Stock cultures were stored at 4 °C on potato dextrose agar (PDA) slants. For enzyme production, the strain was grown in modified Mandels’ salt solution medium supplemented with 1% (w/v) cellulose and 1% (w/v) wheat bran (MMSWC) [19]. The composition of Mandels’ salt solution was as follows: 3 g L^−1^ KH_2_PO_4_, 2.6 g L^−1^ NaNO_3_, 0.5 g L^−1^ urea, 0.5 3 g L^−1^ MgSO_4_·7H_2_O, 0.5 3 g L^−1^ CaCl_2_, 0.0075 g L^−1^ FeSO_4_·7H_2_O, 0.0025 g L^−1^ MnSO_4_·H_2_O, 0.0036 g L^−1^ ZnSO_4_·7H_2_O, 0.0037 g L^−1^ CoCl_2_·6H_2_O, and 1 g L^−1^ peptone.

*Escherichia coli* DH5α (Biomed, Beijing, China) was used as the host for gene cloning, and *P. pastoris* GS115 (Invitrogen, MA, USA) was used for protein expression and was cultured on YPD agar medium (1% yeast extract, 2% peptone, 2% dextrose and 2% agar) at 28 °C. MD medium (2% dextrose, 1.34% YNB, 4 × 10^−5^% biotin and 2% agar) was used to screen the recombinant *P. pastoris*. YPD agar medium containing different concentrations of G418 (geneticin) was used to screen the copy number of the inserted target gene. rPcAxe expression was carried out by inoculating recombinant *P. pastoris* in BMMY medium (1% yeast extract, 2% peptone, 1.34% YNB, 100 mM phosphate buffer pH 6.0, 4 × 10^−5^% biotin and 2% methanol). The *pcaxe* gene was amplified by PCR and the product ligated into the pGEM-T easy vector (Promega, Madison, USA). The pPIC9K vector (Invitrogen, MA, USA) was used for *pcaxe* expression in *P. pastoris*.

### Purification of acetyl xylan esterase

P33 was cultivated in MMSWC medium and fermentation broth was collected on the third day and centrifuged at 4 °C, 8000 rpm for 20 min. The supernatant was salted out with 85% (NH_4_)_2_SO_4_ for 1 h, and the mixture centrifuged at 4 °C, 8000 rpm for 30 min. The pellet was suspended in 10 mM TRIS–HCl buffer (pH 7.5) and dialyzed to remove (NH_4_)_2_SO_4_. The resultant protein solution was loaded onto various columns to isolate the acetyl xylan esterase. The protein solution was loaded on a 1 mL Q Bestarose Fast Flow column (Bestchrom, Shanghai, China) equilibrated in 10 mM TRIS–HCl buffer (pH 7.5), and adsorbed proteins were eluted with a linear concentration gradient of 0–1 M NaCl. The fraction containing acetyl xylan esterase was collected and dialyzed prior to further purification on a Ezload Chromdex 75 Prep Grade column (Bestchrom, Shanghai, China) equilibrated in 10 mM TRIS–HCl buffer (pH 7.5). The active fraction was collected and loaded on a phenyl-sepharose fast flow column (Bestchrom, Shanghai, China), and the adsorbed protein was eluted with a linear concentration gradient of 2–0 M NaCl in 50 mM sodium phosphate (pH 7.5). The active fraction was collected and used for subsequent experiments.

### Amino acid sequencing of the acetyl xylan esterase by LC–MS/MS

The single protein band on the SDS-PAGE gel was cut into small pieces and subjected to tryptic digestion as previously described [20]. The residue was reconstituted with 0.1% formic acid for nanoLC–MS/MS analysis to elucidate the amino acid sequence. The acquired rPcAxe amino acid sequence was used for identification of the signal peptide using the online SignalP tool (http://www.cbs.dtu.dk/services/SignalP/). The sequence was also used in BLAST searches (https://blast.ncbi.nlm.nih.gov/Blast.cgi) to identify related sequences in the database. The rPcAxe sequence was aligned with the extracted reference sequences using MEGA 6.06 software [21] and a neighbor-joining phylogenetic tree was constructed [22]. Domains in rPcAxe were also identified by comparing with the aligned reference sequences.

### Construction of the recombinant plasmid and transformation

The nucleotide sequence of *P. chrysogenum pcaxe* was obtained from the sequence of the most similar protein Pc20g11110. The cDNA of *pcaxe* including the signal peptide was amplified by PCR using forward primer 5′-ATGATGATCCTCCCTGTCC-3′ and reverse primer 5′-TCAAGCCGCCATGAAAAACT-3′ by denaturation at 98 °C for 2 min followed by 30 cycles at 98 °C for 30 s, 55 °C for 30 s, and 72 °C for 1 min, and a final elongation step at 72 °C for 10 min, using Phusion DNA polymerase (New England BioLabs, MA, USA) and the suggested PCR mixture (25 μL). PCR products were purified using a Universal DNA Purification Kit (Tiangen, Beijing, China) and A-tailed using *Taq* DNA polymerase (Thermo Scientific, CA, USA) at 72 °C for 30 min. The A-tailed PCR product was purified using the same method and inserted into the pGEM-T easy vector and transformed into *E. coli* DH5α competent cells. The *pcaxe* gene excluding the signal peptide was amplified by PCR using forward primer 5′-AAGAATTC*CATCATCATCATCATCAT*GCGGCATCTTCGGGCTGCGGC-3′ and reverse primer 5′-AAGCGGCCGCTCAAGCCGCCATGAAAAACTCCCAGGTCGC-3′, in which the underlined sequences indicate restriction sites *Eco*RI and *Not*I that were used for ligation, and letters in italics denoted the His tag for purification of the recombinant protein by affinity chromatography. PCR products were ligated with the plasmid pPIC9K using T4 DNA ligase (Promega, Madison, USA) and transformed into *E. coli* DH5α competent cells.

### Expression and production of recombinant PcAxe

The recombinant pPIC9K-*axe* construct was linearized with the restriction endonuclease *Sal*I, transformed into *P. pastoris* GS115 competent cells by electroporation, and cells were cultured on MD agar medium at 28 °C for 3–4 days. All transformants were scraped and resuspended in sterile water, and grown on YPD agar medium containing different concentrations of G418. The inoculation amount was ~ 10^5^ cells per agar plate, and cultivation proceeded at 28 °C for 2–5 days. The level of protein expression in the transformants was validated in BMMY medium following growth for 72 h with 2% methanol as the inducer.

### Purification of rPcAxe by affinity chromatography

For purification of rPcAxe expressed by *P. pastoris*, the cell-free supernatant was collected by centrifugation at 4 °C, 8000 rpm for 10 min, and filtered through a 0.45 μm filter. The Ni^2+^ His-tag column was equilibrated with binding buffer (20 mM sodium phosphate, 0.5 M NaCl, pH 7.4) and the filtered supernatant was loaded and nonspecific binding proteins removed by wash buffer (20 mM sodium phosphate, 0.5 M NaCl, 20 mM imidazole, pH 7.4). The bound protein was eluted with elution buffer (20 mM sodium phosphate, 0.5 M NaCl, 300 mM imidazole, pH 7.4).

### Enzymatic assays

Acetyl xylan esterase activity was determined using 4-nitrophenyl acetate as the substrate (pNPA) [23]. α-l-Arabinofuranosidase activity was assayed using 4-nitrophenyl-α-l-arabinofuranoside (pNPAF) as the substrate [24]. And β-xylosidase activity measurement was performed using 4-nitrophenyl-β-d-xylopyranoside (pNPX) as the substrate [25]. One unit of enzyme activity was defined as the amount of enzyme required to release 1 μmol of *p*-nitrophenol per min under the conditions assayed.

To determine the optimal pH for acetyl xylan esterase activity, assays were performed at 37 °C in buffers ranging from pH 4–9 using mixtures of 0.1 M citric acid and 0.2 M sodium hydrogen phosphate. The optimal temperature was investigated in the range of 20–70 °C at the optimal pH. The stability of the acetyl xylan esterase at different pH values was determined by measuring the residual activity in standard conditions after incubation of the enzyme in different buffers as described above at room temperature for 1 h. Thermal stability was assessed by measuring the residual activity in standard conditions after incubation of the enzyme at different temperatures for 1 h. Enzyme not pre-incubated was used as a control.

To determine the optimal pH for α-l-arabinofuranosidase activity, the enzyme activity was measured at between pH 5 and 11 using mixtures of 0.1 M citric acid and 0.2 M sodium hydrogen phosphate. The optimal temperature was determined in the range of 20–60 °C at the optimal pH value.

The effect of metal ions (Na^+^, K^+^, Ca^2+^, Fe^3+^, Mg^2+^, Al^3+^, Zn^2+^, Cu^2+^, Pb^2+^, Mn^2+^ and Fe^2+^) and sodium dodecyl sulfate (SDS) on the acetyl xylan esterase activity was determined by incubating the enzyme with the agent at a final concentration of 1, 2, 5 and 10 mM for 1 h at 4 °C. The residual activity was then measured under standard conditions. Enzyme without any added agent was used as a control.

### Substrate specificity and kinetic parameters of the acetyl xylan esterase

The substrate preferences of the purified recombinant acetyl xylan esterase were investigated with 4-hydroxy-3-methoxycinnamic acid methyl ester (a substrate for ferulic acid esterase) and ethyl-4-hydroxy-3-methoxycinnamate and 4-nitrophenyl acetate (pNPA; a substrate for acetyl xylan esterase) using the method described previously [26]. The Michaelis-Menten constant (*K*_m_) and maximal velocity (*V*_max_) were assessed based on pNPA concentrations of 0.2, 0.25, 0.5, 1, 1.25 and 2 mM. *K*_m_ and *V*_max_ were calculated based on the Lineweaver–Burk plot constructed by plotting the reciprocal of the substrate concentration on the x-axis and the reciprocal of the enzyme reaction velocity on the y-axis. All experiments were conducted in triplicate at pH 7 and 40 °C.

### Enzymatic hydrolysis

Corn stover was delignified with sodium chlorite before enzymatic hydrolysis according to the procedure in the Pulp and Paper Technical Association of Canada (PAPTAC) Useful methods G10.U [27]. Enzyme preparations used in the hydrolysis assay consisted of recombinant xylanase from *Schizophyllum commune* (Additional file 1: Figure S1) and cellulase cocktails from *Trichoderma longibrachiatum* (C9748, Sigma, St Louis, MO, USA). Hydrolysis experiments were carried out with 2% (w/v) delignified corn stover in 50 mM sodium acetate buffer (pH 5) in a final reaction volume of 1 mL [20]. For hydrolysis by xylanase, 5 mg protein/g cellulose of rPcAxe was added to the recombinant xylanase (10 mg protein/g cellulose). Reactions were performed in an orbital shaker incubator at 50 °C (the optimal temperature for recombinant xylanase). For hydrolysis by cellulase, varying amounts of commercial cellulase were replaced with the equivalent amount of rPcAxe at a fixed total protein loading (10 mg protein/g cellulose). Reactions were performed in an orbital shaker incubator at 37 °C. Control assays containing enzyme without substrate, and substrate without enzyme, were included under the same conditions. All hydrolysis experiments were conducted in triplicate. Protein concentration was determined using the Bradford Protein Assay Kit (GenStar, Beijing, China) according to the manufacturer’s instructions.

After incubation for the indicated time, hydrolysis was terminated by heating the reaction mixture at 100 °C for 10 min to inactivate the enzymes. The content of total reducing sugar was determined by the DNS method [28]. The individual concentration of glucose and xylose in the supernatants was determined by Essentia LC-15C high performance liquid chromatography (HPLC) (Shimadzu, Kyoto, Japan) using a Remex ROA-organic acid H+ (8%) column (Phenomenex, Los Angeles, CA, USA) and a RID-10A refractive index detector (Shimadzu, Kyoto, Japan). The content of acetic acid was quantified by HPLC using the aforementioned column but with a SPD-15C Essentia UV/VIS detector (Shimadzu, Kyoto, Japan) and the UV wavelength was set at 210 nm. Datasets were tested for statistical significance using ANOVA, and *P* values < 0.05 were deemed significant.

The hydrolysis of wheat arabinoxylan (P-EDWAX30, Megazyme, Wicklow, Ireland) was performed as follows: 400 μL of 1% (w/v) water-soluble wheat arabinoxylan and 100 μL of rPcAxe was reacted in an orbital shaker incubator at 37 °C in buffer mixtures of 0.1 M citric acid and 0.2 M sodium hydrogen phosphate (pH 7.0). After 24 h incubation, reactions were terminated in boiling water for 10 min. Blank controls containing substrate alone were included under the same conditions. The hydrolysates were determined using HPLC according to the method described previously [29].

To examine the synergism between rPcAxe and xylanase on degradation of arabinoxylan, hydrolysis experiments were carried out by adding 2.5 mg protein/g cellulose of rPcAxe into the recombinant xylanase (5 mg protein/g cellulose), with a final reaction volume of 1 mL and a concentration of 1% (w/v) wheat arabinoxylan in 50 mM sodium acetate buffer (pH 5). Reactions were performed in an orbital shaker incubator at 50 °C for 24 h. The contents of total reducing sugar, arabinose and acetic acid were determined by the method described above.

## Results

### Purification and identification of a novel acetyl xylan esterase in *P. chrysogenum* P33

**Fig. 1:**
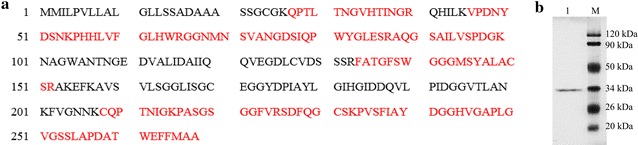
Sequence of PcAxe and SDS-PAGE analysis of purified rPcAxe. **a** Sequence of PcAxe based on LC–MS/MS analysis (the PcAxe band is highlighted in red). **b** SDS-PAGE analysis of purified rPcAxe. Lanes: *M* standard protein molecular weight markers; *1* rPcAxe purified by affinity chromatography

The acetyl xylan esterase was successively purified using strong anion exchange, gel filtration and hydrophobic interaction chromatographic steps. The resultant PcAxe protein ran as a single band on SDS-PAGE with a molecular mass of 31.16 kDa (Additional file 2: Figure S2). The specific activities of purified PcAxe were 4.8 and 0.9 U/mg, with pNPA and pNPAF as the substrates respectively. According to the LC–MS/MS spectrum and database search, PcAxe peptides (Fig. 1a) matched most closely with Pc20g11110, which is annotated as a hypothetical protein in the NCBI database and belongs to CE (carbohydrate esterase) family 1 in the CAZy database.

### Expression and purification of rPcAxe

After induction with methanol at 28 °C for 72 h, the supernatant contained recombinant acetyl xylan esterase activity of 87.46 U/L. Following one-step immobilized metal-affinity chromatography (IMAC), the purified rPcAxe migrated as a single band with an apparent molecular mass of 33.92 kDa in SDS-PAGE (Fig. 1b). The specific activity of purified rPcAxe was 5.4 U/mg with 4-nitrophenyl acetate as the substrate, and 1.2 U/mg with pNPAF as the substrate, which were respectively 12.5 and 33.3% higher than that of the native enzyme.

### Sequence analysis

**Fig. 2:**
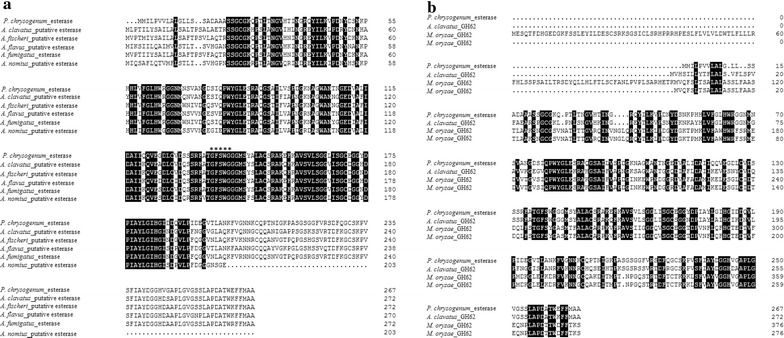
The deduced amino acid sequence alignments of rPcAxe and other esterases and family 62 glycoside hydrolases. **a** The alignment of rPcAxe and other esterases. The consensus motif of the active site of serine peptidases and α/β hydrolases (Gly-Xaa-Ser-Xaa-Gly) was marked with an asterisk. **b** The alignment of rPcAxe and other glycoside hydrolases 62. Aspfi, *Aspergillus fischeri*; Aspfu, *Aspergillus fumigatus*; Aspcl, *Aspergillus clavatus*; Aspfl, *Aspergillus flavus*; Aspno, *Aspergillus nomius*; Magor, *Magnaporthe oryzae*

The nucleotide sequence of *pcaxe* is 804 bp long (Accession Number: KY882362) and corresponds to a protein composed of 267 amino acids. The deduced amino acid alignment showed 99% identity with the Pc20g11110 protein. Although there were three amino acids in rPcAxe that differ from Pc20g11110, the conserved domains of rPcAxe and Pc20g11110 protein were the same. It indicated that rPcAxe and Pc20g11110 protein were identical and performed the same function. A signal peptide of 18 amino acids was also identified in the rPcAxe sequence. By comparing with the reference sequences, it was found that rPcAxe contains an esterase domain and a GH62 domain, which is annotated as α-l-arabinofuranosidase in the CAZy database (Fig. 2). In addition, the Gly-Xaa-Ser-Xaa-Gly consensus motif that is characteristic of the active site of serine peptidases and members of the α/β-hydrolase enzyme family [12] was identified in rPcAxe (Fig. 2). rPcAxe shares high sequence identity with esterases from *Aspergillus flavus* AF70 (77%), *Aspergillus fischeri* NRRL_181 (75%), *Aspergillus fumigatus* Af293 (75%), *Aspergillus clavatus* NRRL_1 (74%) and *Aspergillus nomius* NRRL_13137 (75%). These results imply that rPcAxe is a carboxylesterase belonging to the serine hydrolase superfamily. The sequence identities between rPcAxe and GH62 from *Aspergillus clavatus* NRRL_1, *Magnaporthe oryzae* Y34, *Magnaporthe oryzae* 70–15 and *Penicillium chrysogenum* 31B were 71, 52, 51 and 32% respectively. The neighbor-joining phylogenetic tree containing rPcAxe and the reference sequences (Additional file 3: Figure S3) indicated that rPcAxe is a novel bifunctional enzyme with both esterase and α-l-arabinofuranosidase activities.

### Enzymatic properties of rPcAxe

**Fig. 3:**
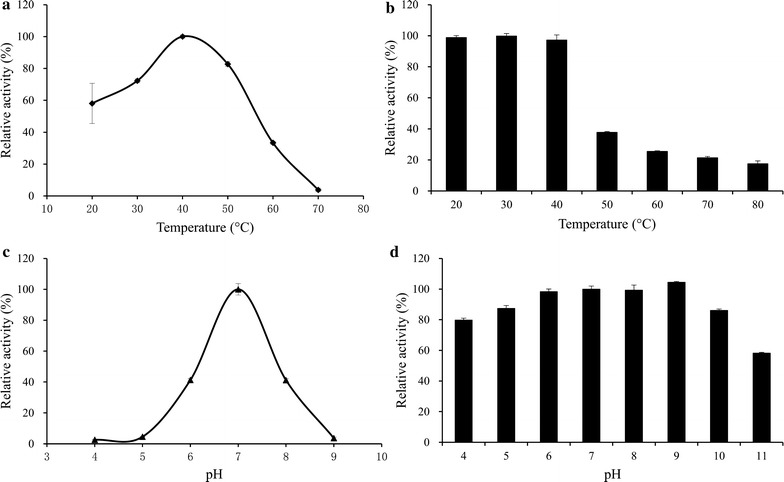
Characterization of purified rPcAxe with 4-nitrophenyl acetate as the substrate. **a** The effect of temperature on enzyme activity. **b** Thermostability of purified rPcAxe. **c** The effect of pH on enzyme activity. Thermal stability was assessed by measuring the residual activity after incubation of rPcAxe at different temperatures for 1 h. **d** pH stability of purified rPcAxe. pH stability was determined by measuring the residual activity after incubation of rPcAxe in different buffers for 1 h. The relative activity was determined, and the maximum activity was defined as 100% (**a**, **c**). The initial activity of rPcAxe not pre-incubated in different buffers was defined as 100% (**b**, **d**). Values are the means and standard deviations of triplicate experiments

The rPcAxe enzyme was unable to hydrolyze 4-hydroxy-3-methoxycinnamic acid methyl ester or ethyl-4-hydroxy-3-methoxycinnamate, but could hydrolyze 4-nitrophenyl acetate and release 4-nitrophenol. The *K*_m_ and the *V*_max_ values for 4-nitrophenyl acetate were 0.465 mM and 0.035 μmol/min/mg, respectively. The purified rPcAxe activity was highest at 40 °C (Fig. 3a), and activity was stable at a temperature range below 40 °C, but the residual activity decreased rapidly at temperature higher than 40 °C (Fig. 3b). The rPcAxe activity was optimal at a pH of 7 (Fig. 3c) and 100% activity was maintained from pH 6 to pH 9 after a 1 h incubation. It could maintain a respective activity of 86 and 58% at pH 10 and pH 11 after a 1 h incubation, indicating strong pH stability (Fig. 3d).

The rPcAxe activity was slightly enhanced in the presence of Mg^2+^, Cu^2+^ and Pb^2+^ at a concentration of 5 mM, and by Mn^2+^ and Fe^2+^ at 10 mM, with relative activities ranging from 102.1 to 109.0%. The relative activity of rPcAxe was maintained at 95.8 and 92.7% in the presence of 5 mM Mn^2+^ and Fe^2+^. The ions Na^+^, K^+^, Ca^2+^, Fe^3+^, Al^3+^ and Zn^2+^ had a minor inhibitory effect under different concentrations, as did the detergent SDS (Table 1). These results indicate that rPcAxe is relatively stable in the presence of these metal ions.

**Table 1:** Effect of metal ions and chemical reagents on the enzyme activity of rPcAxe

Metal ions	Relative activity (%)^a^			
	1 mM	2 mM	5 mM	10 mM
Na^+^	94.1 ± 1.0	98.9 ± 2.1	97.9 ± 3.5	87.2 ± 4.5
K^+^	91.0 ± 1.1	91.0 ± 1.4	93.3 ± 0.7	88.6 ± 2.1
Ca^2+^	89.9 ± 2.0	95.5 ± 1.6	96.5 ± 1.5	86.7 ± 2.9
Fe^3+^	89.1 ± 1.5	89.7 ± 0.7	87.9 ± 2.3	76.9 ± 1.0
Mg^2+^	89.8 ± 1.6	90.9 ± 1.9	102.1 ± 1.6	93.6 ± 5.9
Al^3+^	92.6 ± 2.1	93.7 ± 2.2	87.8 ± 5.0	82.6 ± 1.6
Zn^2+^	88.4 ± 3.2	90.5 ± 1.3	91.3 ± 3.5	89.7 ± 3.8
Cu^2+^	98.6 ± 1.8	97.4 ± 1.7	108.8 ± 1.4	96.8 ± 2.0
Pb^2+^	94.0 ± 1.3	95.4 ± 2.1	109.0 ± 4.2	102.3 ± 0.8
Mn^2+^	86.4 ± 1.4	92.1 ± 3.1	95.8 ± 2.0	103.3 ± 3.0
Fe^2+^	82.5 ± 4.2	93.5 ± 2.2	92.7 ± 6.5	105.7 ± 2.2
SDS	85.0 ± 1.7	90.6 ± 2.5	88.7 ± 1.5	72.7 ± 2.3

^a^Values represent the mean ± SD (n = 3) relative to untreated control samples

### α-l-Arabinofuranosidase activity of rPcAxe

**Fig. 4:**
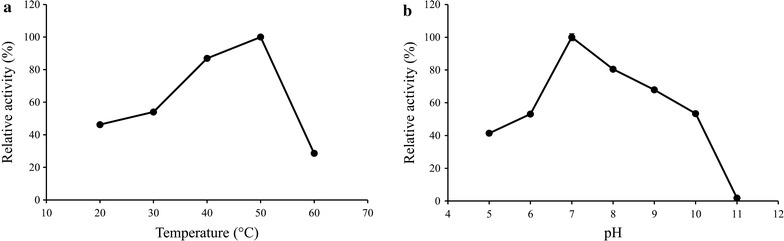
Effects of pH and temperature on the enzyme activity of purified rPcAxe with 4-nitrophenyl-α-l-arabinofuranoside. **a** Effect of temperature on enzyme activity. The optimal temperature was determined in the range of 20–60 °C. **b** Effect of pH on enzyme activity. Enzyme activity was measured between pH 5 and 11 using mixtures of 0.1 M citric acid and 0.2 M sodium hydrogen phosphate. The maximum activity was defined as 100%, and the relative activity was determined. Values are the means and standard deviations of triplicate experiments

Based upon its high level of sequence identity shared with GH62 (Fig. 2), the α-l-arabinofuranosidase activity of rPcAxe was tested using 5 mM pNPAF as the substrate. The optimal pH and temperature were 7.0 and 50 °C, respectively (Fig. 4). The relative activity for arabinofuranosidase was 87% at 40 °C, which was the optimal temperature for acetyl xylan esterase.

**Fig. 5:**
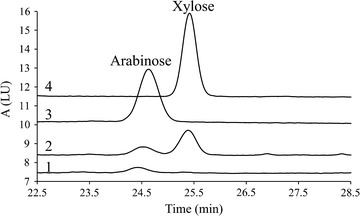
Hydrolysis of water-soluble wheat arabinoxylan by purified rPcAxe. 1, Substrate incubated without enzyme for 24 h (control); 2, hydrolysate treated with rPcAxe for 24 h; 3, arabinose standard; 4, xylose standard. Hydrolysis reactions were performed at 37 °C in buffer consisting of mixtures of 0.1 M citric acid and 0.2 M sodium hydrogen phosphate (pH 7.0) for 24 h with 1% (w/v) water-soluble wheat arabinoxylan loading. The amount of arabinose and xylose released was determined using HPLC. Values are the means and standard deviations of triplicate experiments

In order to confirm the α-l-arabinofuranosidase activity of rPcAxe, we examined the action of rPcAxe on wheat arabinoxylan. The optimal temperature for the α-l-arabinofuranosidase activity towards pNPAF of rPcAxe was 50 °C (Fig. 4). However, the thermostability of the α-l-arabinofuranosidase activity of rPcAxe at 37 °C was higher than that at 50 °C (Additional file 4: Figure S4). The relative α-l-arabinofuranosidase activity was 80% at 37 °C (Fig. 4), hence the hydrolysis of wheat arabinoxylan was performed at this temperature. After 24 h of hydrolysis, rPcAxe released 3.16 μg/mL arabinose from wheat arabinoxylan, which was 0.13% of the total arabinose in the substrate. Surprisingly, 21.22 μg/mL xylose was also released in addition to arabinose (Fig. 5), indicating that rPcAxe could cleave the β-1,4 bonds in the xylan backbone. Purified rPcAxe was therefore used to further validate the β-xylosidase activity, and a specific activity of 0.512 U/mg with 4-nitrophenyl-β-d-xylopyranoside as the substrate was observed.

### Action of rPcAxe in enzymatic hydrolysis

**Fig. 6:**
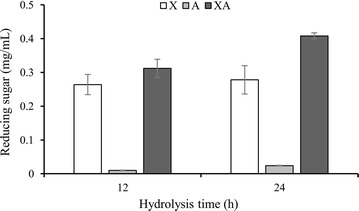
Total reducing sugars released from delignified corn stover by recombinant xylanase, rPcAxe and the enzymatic mix. Hydrolysis reactions were performed at 50 °C for 12 or 24 h with 2% (w/v) biomass loading. The amount of reducing sugars released was determined using the DNS method. X (white bars), recombinant xylanase from *S. commune*; A (light gray bars), rPcAxe; XA (dark gray bars), mixtures of recombinant xylanase from *S. commune* and rPcAxe. Values are the means and standard deviations of triplicate experiments

In order to investigate the effects of rPcAxe on the hydrolysis of lignocellulose, hydrolysis of delignified corn stover was performed in combination with recombinant xylanase or commercial cellulase. After 24 h of hydrolysis, the released reducing sugar content was 0.278 and 0.408 mg/mL with recombinant xylanase alone and with recombinant xylanase supplemented with rPcAxe (Fig. 6). The degree of synergy between rPcAxe and xylanase was calculated as 1.35 at 24 h.

**Fig. 7:**
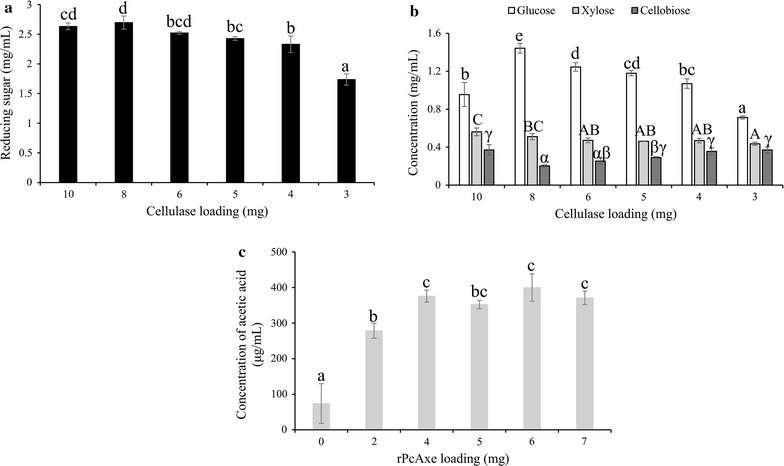
Hydrolysis of delignified corn stover using cellulase and rPcAxe at a fixed total protein dosage. **a** Amount of released reducing sugars determined by the DNS method. **b** Amount of glucose (white bars), xylose (light gray bars) and cellobiose (dark gray bars) determined by HPLC. **c** Concentration of acetic acid in the hydrolysate determined by HPLC. Hydrolysis reactions were performed at 37 °C for 24 h with 2% (w/v) biomass loading. Values are the means and standard deviations of triplicate experiments. Statistical significance is indicated by different letters on columns based on ANOVA (*P* < 0.05)

The effects of rPcAxe on cellulose hydrolysis were also investigated and are presented in Fig. 7. The amount of released reducing sugars was 2.5% higher with a mixture containing 8 mg of commercial cellulase and 2 mg of rPcAxe than with 10 mg of commercial cellulase alone. When 4 or 5 mg of commercial cellulase was replaced by the equivalent amount of rPcAxe, the released reducing sugar content was 2.52 and 2.43 mg/mL, respectively, which was not significantly different from that obtained using 10 mg of commercial cellulase alone (*P* < 0.05; Fig. 7a). HPLC analysis revealed that the amount of glucose released by a mixture of 8 mg cellulase and 2 mg rPcAxe was 51% higher than that released by cellulase alone. The enzyme mixture of 8 mg cellulase and 2 mg rPcAxe released 14.1% of the total glucose in the substrate. Meanwhile, the amount of cellobiose released was 45% lower in the mixture. In the treatment containing 5 mg cellulase and 5 mg rPcAxe, the amount of glucose released was significantly higher (*P* < 0.05) than that released by cellulase alone (Fig. 7b). An increase in the concentration of acetic acid was also observed when a higher proportion of commercial cellulase was replaced by purified rPcAxe, and the highest concentration of acetic acid was obtained when up to 60% of commercial cellulase was replaced. These results confirmed that rPcAxe could act on the acetyl esters of delignified corn stover (Fig. 7c).

The synergism between rPcAxe and the recombinant xylanase on degradation of arabinoxylan was also examined (Additional file 5: Table S1). After 24 h of hydrolysis, the reducing sugar contents released by recombinant xylanase alone and by its synergy with rPcAxe were 0.423 and 0.665 mg/mL respectively, and the degree of synergism was 1.454. When arabinoxylan was hydrolyzed by rPcAxe alone, 0.13% of the total arabinose in the substrate was released (0.003 mg/mL). In comparison, by the synergistic hydrolysis of rPcAxe and recombinant xylanase, the released arabinose increased by 200%, and the released acetic acid went up by 25% (0.057–0.071 mg/mL).

## Discussion

Cellulose and hemicellulose chains form an alternating layered structure in the cell wall, and the presence of hemicellulose hinders the hydrolysis of cellulose [4]. Acetyl xylan esterase is an important auxiliary enzyme that remove acetyl groups from the side chains of hemicellulose, promoting the hydrolysis of hemicellulose and cellulose [30]. In the present study, the *K*_m_ of rPcAxe was 0.465 mM, which was higher than that of the acetyl xylan esterase from *P. chrysosporium* [12], but lower than that from *Aspergillus awanori* [31]. The *V*_max_ of rPcAxe with pNPA as the substrate was lower than that of the acetyl xylan esterase from *Coprinopsis cinerea* [32]. rPcAxe displayed optimal acetyl xylan esterase activity at pH 7, similar to other fungal acetyl xylan esterases such as those from *P. chrysosporium* [12], *Aspergillus ficuum* [33] and *A. awamori* [31]. However, the stable activity between pH 4 and pH 9, especially the retention of full enzyme activity after incubation between pH 6 and 9 for 1 h, marked rPcAxe as different from all other previously described fungal acetyl xylan esterases. During incubation of strain P33, the pH value of the culture increased gradually and reached ~ 8.1, during which the acetyl xylan esterase activity remained high (data not shown), in accordance with the basophilic properties of rPcAxe. Industrial processes often include steps at different pH, and the majority of known enzymes need to be stabilized under such conditions. Therefore, enzymes that are inherently stable under these conditions have great potential in industrial applications [34, 35]. It was reported that the rPcAxe2 enzyme from *P. chrysosporium* lost more than half of its relative activity in the presence of Mn^2+^ and Fe^2+^ (5 mM), and activity dropped to 1.6% when incubated with 5 mM Fe^2+^ [12]. In our study, the relative activity of rPcAxe was maintained at 95.8 and 92.7% in the presence of 5 mM Mn^2+^ and Fe^2+^, respectively. rPcAxe could maintain a relative activity of more than 80% with the presence of most of the tested metal ions except Fe^3+^ (10 mM). This resistance to metal ions is also advantageous in industrial applications, since metal ions are inevitably present in industrial production. rPcAxe displayed optimal α-l-arabinofuranosidase activity at pH 7, which is typical for bacterial arabinofuranosidases [24, 36], and the same was true for its acetyl xylan esterase activity, which differs from PcAxe2 from *P. chrysosporium* [12] and most other fungal arabinofuranosidases (optimal pH less than 5.0) [37]. These characteristics make rPcAxe a promising enzyme for biotechnological use.

In the hydrolysis of delignified corn stover, a significant synergistic effect (*P* < 0.05) was observed in the mixture of rPcAxe and the recombinant xylanase (degree of synergism = 1.35). This suggested that rPcAxe promoted the hydrolytic activity of xylanase. Although the total reducing sugar production was almost the same in a mixture of 8 mg of commercial cellulase and 2 mg of rPcAxe as that with 10 mg of cellulase alone, the release of glucose was increased by 51%, and the amount of cellobiose in the mixture was reduced, suggesting rPcAxe markedly promoted the hydrolysis of cellulose. It was speculated that the removal of ester groups by rPcAxe enhanced the β-glucosidase activity in the commercial cellulase cocktail, which hydrolyzed the cellobiose to liberate more glucose, although it was not directly involved in the hydrolysis of cellulosic component. Previous studies showed similar results. The amount of glucose released from pretreated wheat straw by the combination of cellulase and xylanase plus acetyl xylan esterase was higher than that released by the combination of cellulase and xylanase, while the amount of cellobiose was lower [4]. When the dosage of commercial cellulase was decreased, the decrease in released xylose was not significant. Because rPcAxe cleaves β-1,4 bonds in the xylan backbone (Fig. 5), it is likely that rPcAxe releases xylose, which presumably made up for the decrease in xylose resulting from the use of less commercial cellulase. This showed that rPcAxe could facilitate the hydrolytic activity of xylanase in the commercial cellulase cocktail [20]. It is likely that the content of arabinose in delignified corn stover was too low to be detected in the enzymatic hydrolysis assays. Partially replacing commercial cellulase with an equivalent amount of rPcAxe markedly increased the release of glucose, and promoted the hydrolysis of cellulose. These results suggest increasing the dosage of cellulase was not necessary for effective lignocellulose hydrolysis, and hemicellulolytic enzymes appeared to play an important role in biomass degradation. Zhu et al. [38] reported a high degree of synergism (up to 75%) between a commercial fungal cellulase and a hemicellulase-enriched mixture derived from a bacterial consortia upon replacement of the commercial enzymes. Synergy between cellulolytic and hemicellulolytic enzymes and acetyl xylan esterase has been studied widely [4, 12, 30, 39, 40], and it is a useful way to increase the sugar yield and reduce the enzyme cost. The presence of acetyl groups prevents cellulase from accessing cellulose [4]. It was speculated that rPcAxe hydrolyzes the ester groups connected to hemicellulose, thereby creating new hydrolytic sites for xylanase and increasing the accessibility of xylan to xylanase, and subsequently increasing the accessibility of cellulose to cellulase [4, 41].

In the present study, we observed activity against water-soluble wheat arabinoxylan, as demonstrated by the release of arabinose and acetic acid after 24 h of hydrolysis, proving that rPcAxe possessed α-l-arabinofuranosidase activity in addition to acetyl xylan esterase activity. rPcAxe in this study was active on pNPAF and arabinoxylan. It mainly displayed acetyl xylan esterase activity, and the activity towards arabinoxylan was low. Bifunctional enzymes showed one main enzyme activity, which was similar to what had been reported by Yang et al. [5]. The structure and composition of lignocellulose is complicated. Therefore, to effectively degrade lignocellulose, fungi usually secrete a set of extracellular lignocellulolytic enzymes [42], and anaerobic bacteria have evolved cell-associated multiprotein complexes known as the cellulosome [43] or xylosome [44]. Another important strategy in lignocellulolytic microbes is the induction of multifunctional enzymes to degrade different substrates [7], and this has received significant attention of late [5, 6, 45]. To our knowledge, the present work is the first detailed report of a bifunctional enzyme possessing acetyl xylan esterase and α-l-arabinofuranosidase activities. Acetyl xylan esterase and α-l-arabinofuranosidase are both important auxiliary enzymes. This bifunctional enzyme must be combined with cellulolytic/hemicellulolytic enzymes (e.g. cellulases or hemicellulases) to effectively degrade lignocellulose. Due to the bifunctionality of rPcAxe, the mixture of enzymes needed for effective lignocellulose hydrolysis can be reduced, which can lower the enzyme cost. Additionally, to a certain extent, the problems associated with the complex interactions and the difficulty in controlling different glycoside hydrolases can be avoided, and the efficiency of enzymatic hydrolysis improved. We anticipate being able to design simpler and lower-cost xylanolytic cocktails in the future based on the discovery of rPcAxe.

## Conclusions

The novel bifunctional enzyme PcAxe was identified in *P. chrysogenum* P33. rPcAxe includes a carbohydrate esterase domain and a glycosyl hydrolase family 62 domain. rPcAxe is stable across a broad pH range, retaining 100% enzyme activity om pH 6–9 after a 1 h incubation. The enzyme tolerates the presence of a wide range of metal ions, and a significant synergy with xylanase was apparent; rPcAxe enhanced the hydrolysis of cellulose by hydrolyzing side groups, and improved the glucose yield. These findings expand our current knowledge of glycoside hydrolases and pave the way for the discovery of similar novel enzymes.

## Additional files

**Additional file 1: Figure S1.** Recombinant xylanase from *S. commune*. Lanes: M, standard protein molecular weight markers; rScXYL, recombinant xylanase from *S. commune* purified by affinity chromatography. The arrow indicates recombinant xylanase.

**Additional file 2: Figure S2.** Elution profiles of acetyl xylan esterase from *P. chrysogenum* P33 and SDS-PAGE analysis of purified PcAxe. a, Ion exchange chromatography. b, Gel filtration chromatography. c, Hydrophobic interaction chromatography. d, SDS-PAGE analysis of purified PcAxe. Lanes: M, standard protein molecular weight markers; AXE, purified PcAxe. The arrow indicates purified PcAxe.

**Additional file 3: Figure S3.** Phylogenetic tree of rPcAxe. The phylogenetic tree was constructed by the neighbor-joining method using MEGA 6.06 software. Aspfi, *Aspergillus fischeri*; Aspfu, *Aspergillus fumigatus*; Aspcl, *Aspergillus clavatus*; Aspfl, *Aspergillus flavus*; Aspno, *Aspergillus nomius*; Magor, *Magnaporthe oryzae*.

**Additional file 4: Figure S4.** Thermostability of purified rPcAxe with 4-nitrophenyl-α-L-arabinofuranoside as the substrate. Thermal stability was assessed by measuring the residual activity after incubation of rPcAxe at different temperatures for 1 h. The initial activity of rPcAxe not pre-incubated in different buffers was defined as 100%. Values are the means and standard deviations of triplicate experiments.

**Additional file 5: Table S1.** Hydrolysis of water-soluble wheat arabinoxylan by recombinant xylanase, rPcAxe and the enzymatic mix.

## Abbreviations

CAZy: carbohydrate-active enzyme database
CE: carbohydrate esterase
DNS: 3,5-dinitrosalicylic acid
GH: glycoside hydrolase
PDA: potato dextrose agar
HPLC: high performance liquid chromatography
IMAC: immobilized metal-affinity chromatography
*K*_m_: Michaelis–Menten constant
*V*_max_: maximal velocity
MMSWC: modified Mandels’ salt solution supplemented with 1% (w/v) cellulose plus 1% (w/v) wheat bran
nanoLC–MS/MS: nano liquid chromatography–tandem mass spectrometry
PcAxe: acetyl xylan esterase from *Penicillium chrysogenum* P33
rPcAxe: recombinant PcAxe
pNPA: 4-nitrophenyl acetate
pNPAF: 4-nitrophenyl-α-l-arabinofuranoside
pNPX: 4-nitrophenyl-β-d-xylopyranoside
SDS: sodium dodecyl sulfate
SDS-PAGE: sodium dodecyl sulfate polyacrylamide gel electrophoresis
