# Supplementary figures and images for: A novel bifunctional acetyl xylan esterase/arabinofuranosidase from Penicillium chrysogenum P33 enhances enzymatic hydrolysis of lignocellulose
Source: Microb Cell Fact. 2017 Sep 26;16:166. doi: 10.1186/s12934-017-0777-7 (PMC5615437; doi:10.1186/s12934-017-0777-7)

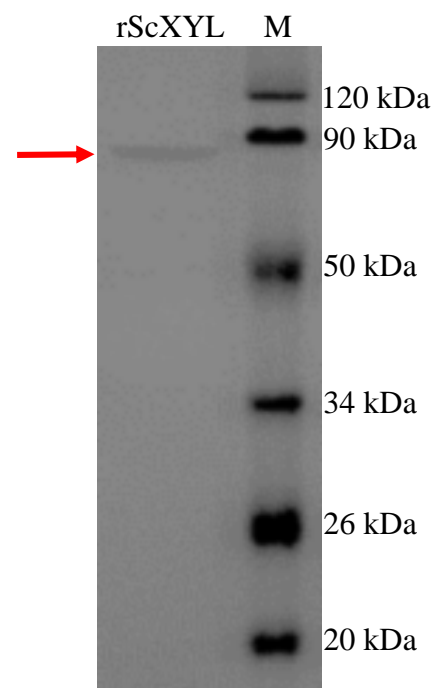

Supplement: Supplementary file 1 — Additional file 1: Figure S1. Recombinant xylanase from S. commune. Lanes: M, standard protein molecular weight markers; rScXYL, recombinant xylanase from S. commune purified by affinity chromatography. The arrow indicates recombinant xylanase. [file 12934_2017_777_MOESM1_ESM.pdf]

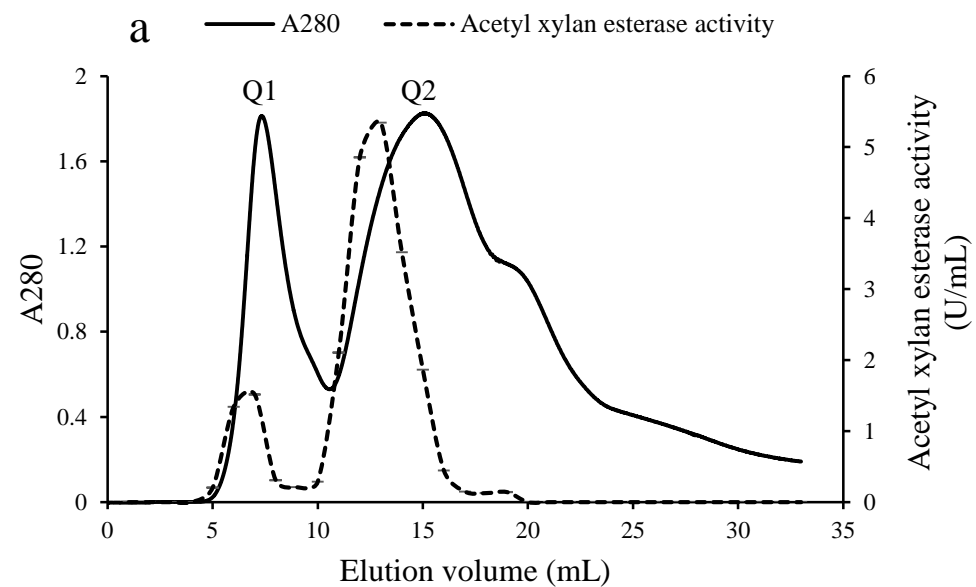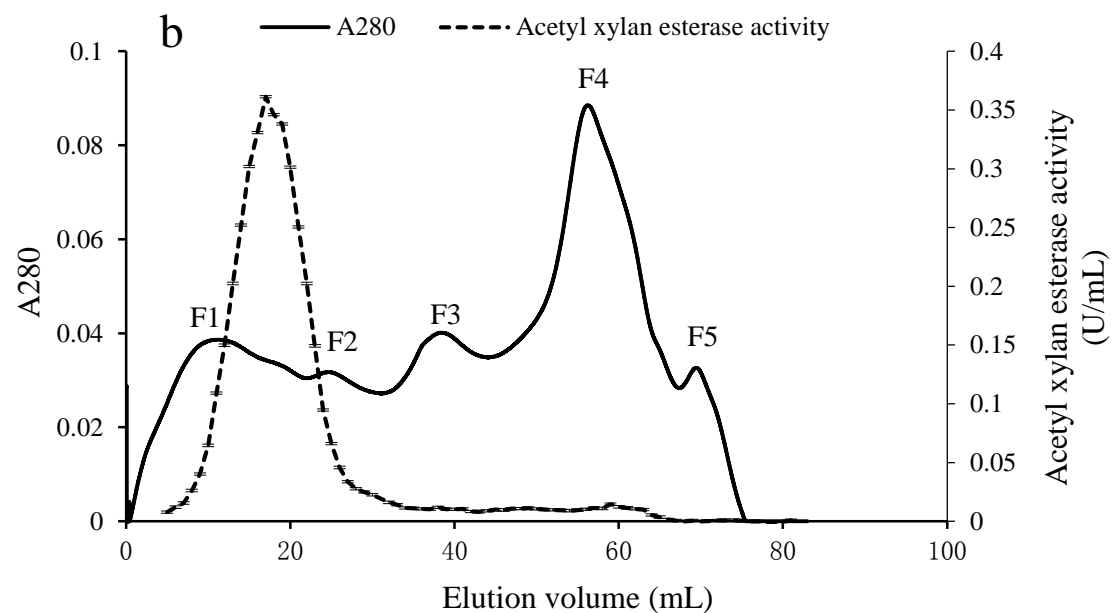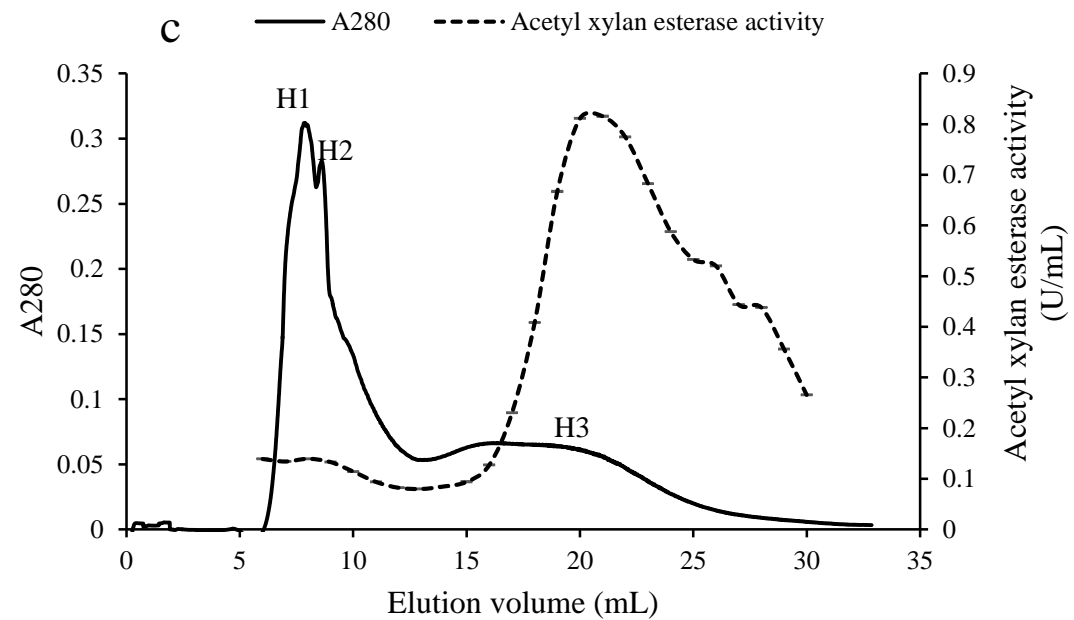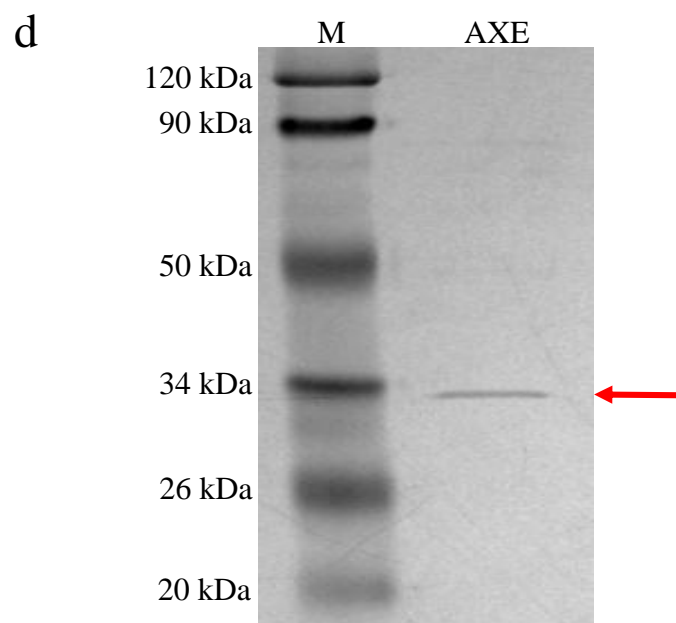

Supplement: Supplementary file 2 — Additional file 2: Figure S2. Elution profiles of acetyl xylan esterase from P. chrysogenum P33 and SDS-PAGE analysis of purified PcAxe. a, Ion exchange chromatography. b, Gel filtration chromatography. c, Hydrophobic interaction chromatography. d, SDS-PAGE analysis of purified PcAxe. Lanes: M, standard protein molecular weight markers; AXE, purified PcAxe. The arrow indicates purified PcAxe. [file 12934_2017_777_MOESM2_ESM.pdf]

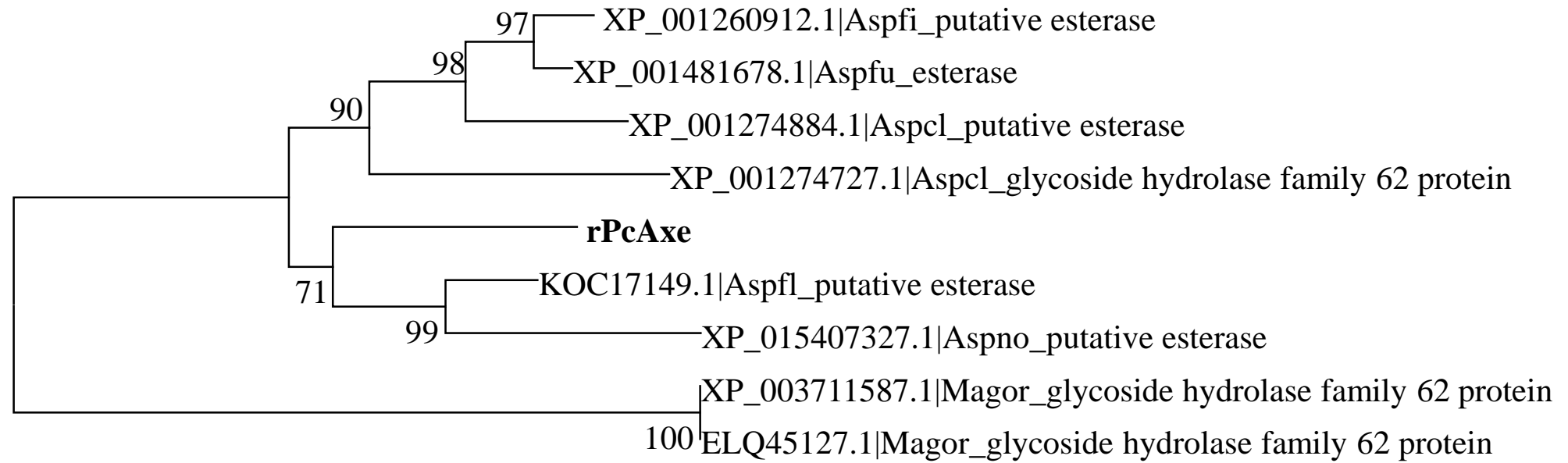

0.05

Supplement: Supplementary file 3 — Additional file 3: Figure S3. Phylogenetic tree of rPcAxe. The phylogenetic tree was constructed by the neighbor-joining method using MEGA 6.06 software. Aspfi, Aspergillus fischeri; Aspfu, Aspergillus fumigatus; Aspcl, Aspergillus clavatus; Aspfl, Aspergillus flavus; Aspno, Aspergillus nomius; Magor, Magnaporthe oryzae. [file 12934_2017_777_MOESM3_ESM.pdf]

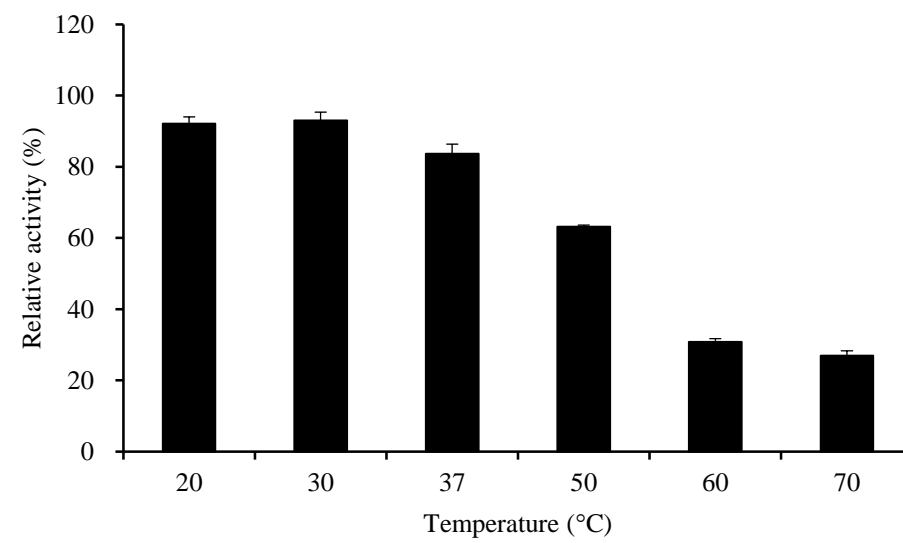

Supplement: Supplementary file 4 — Additional file 4: Figure S4. Thermostability of purified rPcAxe with 4-nitrophenyl-α-L-arabinofuranoside as the substrate. Thermal stability was assessed by measuring the residual activity after incubation of rPcAxe at different temperatures for 1 h. The initial activity of rPcAxe not pre-incubated in different buffers was defined as 100%. Values are the means and standard deviations of triplicate experiments. [file 12934_2017_777_MOESM4_ESM.pdf]
