# Supplementary material for: A novel bifunctional acetyl xylan esterase/arabinofuranosidase from Penicillium chrysogenum P33 enhances enzymatic hydrolysis of lignocellulose
Source: Microb Cell Fact. 2017 Sep 26;16:166. doi: 10.1186/s12934-017-0777-7 (PMC5615437; doi:10.1186/s12934-017-0777-7)
Supplement: Supplementary file 5 — Additional file 5: Table S1. Hydrolysis of water-soluble wheat arabinoxylan by recombinant xylanase, rPcAxe and the enzymatic mix. [file 12934_2017_777_MOESM5_ESM.pdf]

Table S1 Hydrolysis of water-soluble wheat arabinoxylan by recombinant xylanase, rPcAxe and the enzymatic mix

Enzyme	Reducing sugar (mg/mL)	Xylose (mg/mL)	Arabinose (mg/mL)	Acetic acid (mg/mL)
X [a]	0.423 ± 0.007	0.295 ± 0.003	ND [d]	ND
A [b]	0.034 ± 0.009	0.029 ± 0.001	0.003 ±0.001	0.057 ± 0.004
XA [c]	0.665 ± 0.017	0.507 ± 0.012	0.009 ±0.001	0.071 ± 0.002

Values are the means and standard deviations of triplicate experiments.

[a] Recombinant xylanase from *S. commune*;

[b] rPcAxe;

[c] Mixtures of recombinant xylanase from *S. commune* and rPcAxe;

[d] Not detected.
